# Damage-tolerant nanotwinned metals with nanovoids under radiation environments

**DOI:** 10.1038/ncomms8036

**Published:** 2015-04-24

**Authors:** Y. Chen, K Y. Yu, Y. Liu, S. Shao, H. Wang, M. A. Kirk, J. Wang, X. Zhang

**Affiliations:** 1 Department of Materials Science and Engineering, Texas A&M University, College Station, Texas 77843, USA; 2 Department of Materials Science and Engineering, China University of Petroleum-Beijing, Beijing 102246, China; 3 MST-8, Los Alamos National Laboratory, Los Alamos, New Mexico 87545, USA; 4 Department of Electrical and Computer Engineering, Texas A&M University, College Station, Texas 77843, USA; 5 Nuclear Engineering Division, Argonne National Laboratory, Argonne, Illinois 60439, USA; 6 Department of Mechanical and Materials Engineering, University of Nebraska-Lincoln, Lincoln, Nebraska 68588, USA; 7 Department of Mechanical Engineering, Texas A&M University, College Station, Texas 77843-3123, USA

## Abstract

Material performance in extreme radiation environments is central to the design of future nuclear reactors. Radiation induces significant damage in the form of dislocation loops and voids in irradiated materials, and continuous radiation often leads to void growth and subsequent void swelling in metals with low stacking fault energy. Here we show that by using *in situ* heavy ion irradiation in a transmission electron microscope, pre-introduced nanovoids in nanotwinned Cu efficiently absorb radiation-induced defects accompanied by gradual elimination of nanovoids, enhancing radiation tolerance of Cu. *In situ* studies and atomistic simulations reveal that such remarkable self-healing capability stems from high density of coherent and incoherent twin boundaries that rapidly capture and transport point defects and dislocation loops to nanovoids, which act as storage bins for interstitial loops. This study describes a counterintuitive yet significant concept: deliberate introduction of nanovoids in conjunction with nanotwins enables unprecedented damage tolerance in metallic materials.

## Abstract (web summary)

Materials that are tolerant to radiation damage are important for use in nuclear reactors or the storage of nuclear materials. Here, the authors show that pre-introduced nanovoids in nanotwinned copper improve self-healing of the material and therefore enhance its radiation tolerance.

## Graphical abstract

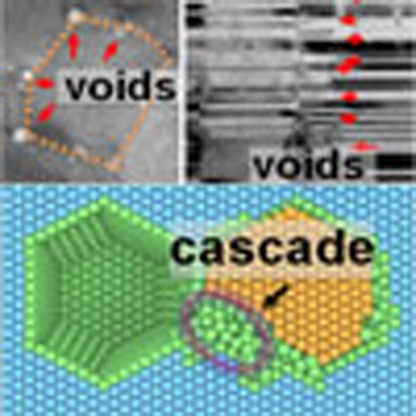

Materials that can sustain extreme environments, such as high stress and radiation, are constantly being sought for unprecedented performance. Nuclear energy currently accounts for more than 10% of electricity world wide, and the discovery of advanced materials for extreme radiation environments resides at the centre of the design of future nuclear reactors^1^,^2^,^3^,^4^,^5^,^6^. Irradiation of metals by neutrons or heavy ions results in a large number of point defects^7^,^8^,^9^ and their clusters, including dislocation loops, voids and stacking fault tetrahedra^10^,^11^,^12^,^13^,^14^,^15^, which cause severe void swelling, radiation hardening, embrittlement and creep^16^,^17^,^18^. Interfacial defect sinks, such as grain boundaries^19^,^20^,^21^,^22^,^23^, heterophase interfaces^24^,^25^,^26^,^27^,^28^ and free surfaces^29^,^30^,^31^, have proven to be effective in alleviating radiation damage. Grain size dependence of void swelling in stainless steel was observed previously^19^ and recieved renewed intense interest as nanograins appear to drastically enhance radiation tolerance as shown experimentally^21^,^23^ and theoretically^20^,^32^,^33^,^34^,^35^. However, nanograins tend to coarsen at elevated temperature and under irradiation^3^,^36^, compromising their radiation tolerance. Recent studies show that nanotwinned (nt) metals have high strength and ductility^37^,^38^,^39^,^40^,^41^,^42^, outstanding microstructural stability under both radiation^43^ and annealing conditions^44^,^45^, and twin boundaries (TBs) serve as defect sinks and destruct stacking fault tetrahedra in nt metals^46^,^47^.

In general, continuous intense radiation leads to high-density voids with increasing void size and void swelling in irradiated metallic materials. However, here we show that by deliberate combination of nanovoids with nanotwins, unprecedented radiation tolerance could be achieved in irradiated nanovoid-nanotwinned (nv-nt) Cu. Using *in situ* radiation inside a transmission electron microscope, we observed self-healing of nanovoids. Atomistic simulations reveal that nanotwins are essential to achieve superior radiation tolerance as TB networks consisting of coherent TBs (CTBs) and incoherent TBs (ITBs) promote rapid migration of defect clusters to nanovoids, wherein they are annihilated. Nanovoids act as defect sinks to absorb radiation-induced interstitial loops, as revealed by *in situ* radiation and confirmed by molecular dynamics (MD) simulations. This study provides a fresh perspective on the design of metallic materials with extraordinary damage tolerance.

## Results

### TB and nanovoid morphology in nv-nt Cu

**Figure 1:**
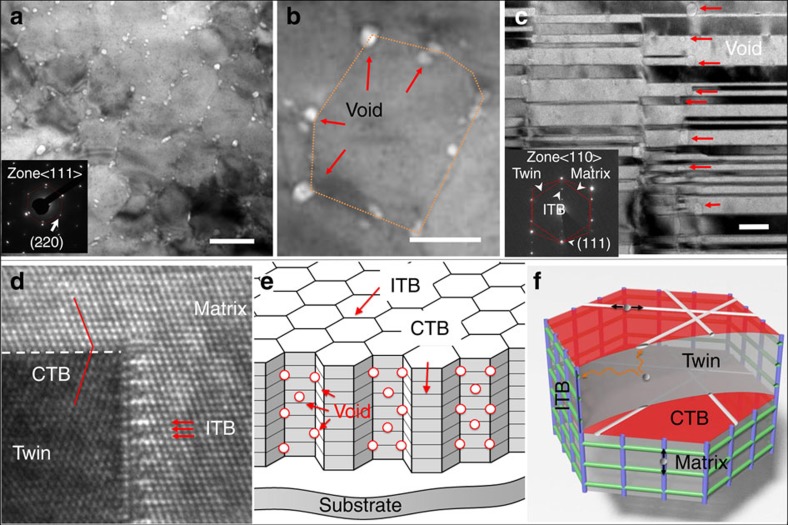
Deliberate introduction of nanovoids and nanotwins in Cu (nv-nt Cu). (**a**–**b**) Plan-view transmission electron microscopy (TEM) micrograph showing the as-prepared nv-nt Cu film containing abundant nanovoids primarily surrounding columnar domain boundaries. Scale bar (**a**), 100 nm; scale bar (**b**), 50 nm. (**c**) Cross-section TEM micrograph shows high-density Σ 3{111} coherent twin boundaries (CTB) with an average twin thickness of ∼15 nm, and Σ 3{112} incoherent twin boundaries (ITBs), which were decorated by a large number of nanovoids with an average diameter of∼10 nm. The inserted selected area diffraction (SAD) pattern confirms the formation of epitaxial nt Cu. Scale bar, 20 nm. (**d**) High-resolution TEM image of CTBs and ITBs. (**e**) A conceptual schematic of metals with CTB and ITB networks and nanovoids. (**f**) Inside a typical columnar grain radiation-induced interstitials or their loops can rapidly migrate towards ITBs, where they can migrate rapidly to nanovoids.

Plan-view transmission electron microscopy (TEM) micrograph shows the as-prepared Cu contained abundant nanovoids primarily surrounding columnar domain boundaries (Fig. 1a,b) (see Supplementary Fig. 1). Figure 1c shows high-density CTBs with an average twin thickness of ∼15 nm, and ITBs that were decorated by a large number of nanovoids with an average diameter of ∼10 nm. These three-dimensional voids distributed at different depth in the film are introduced during magnetron sputtering process, and void density can be controlled by tailoring deposition rate, substrate temperature as well as epitaxy between film and substrate. High-resolution TEM image in Fig. 1d shows atomic structure of CTBs and ITBs. Figure 1e displays a conceptual schematic of nv-nt metals that contain ITB-CTB networks and nanovoids along ITBs. Figure 1f shows diffusion channels associated with dislocations at CTBs and ITBs that could potentially transport interstitials and their clusters towards nanovoids. The significance of such ITB-CTB networks on radiation response of nt metals will be revisited later.

### Irradiation-induced morphology evolution in nv-nt and cg Cu

**Figure 2:**
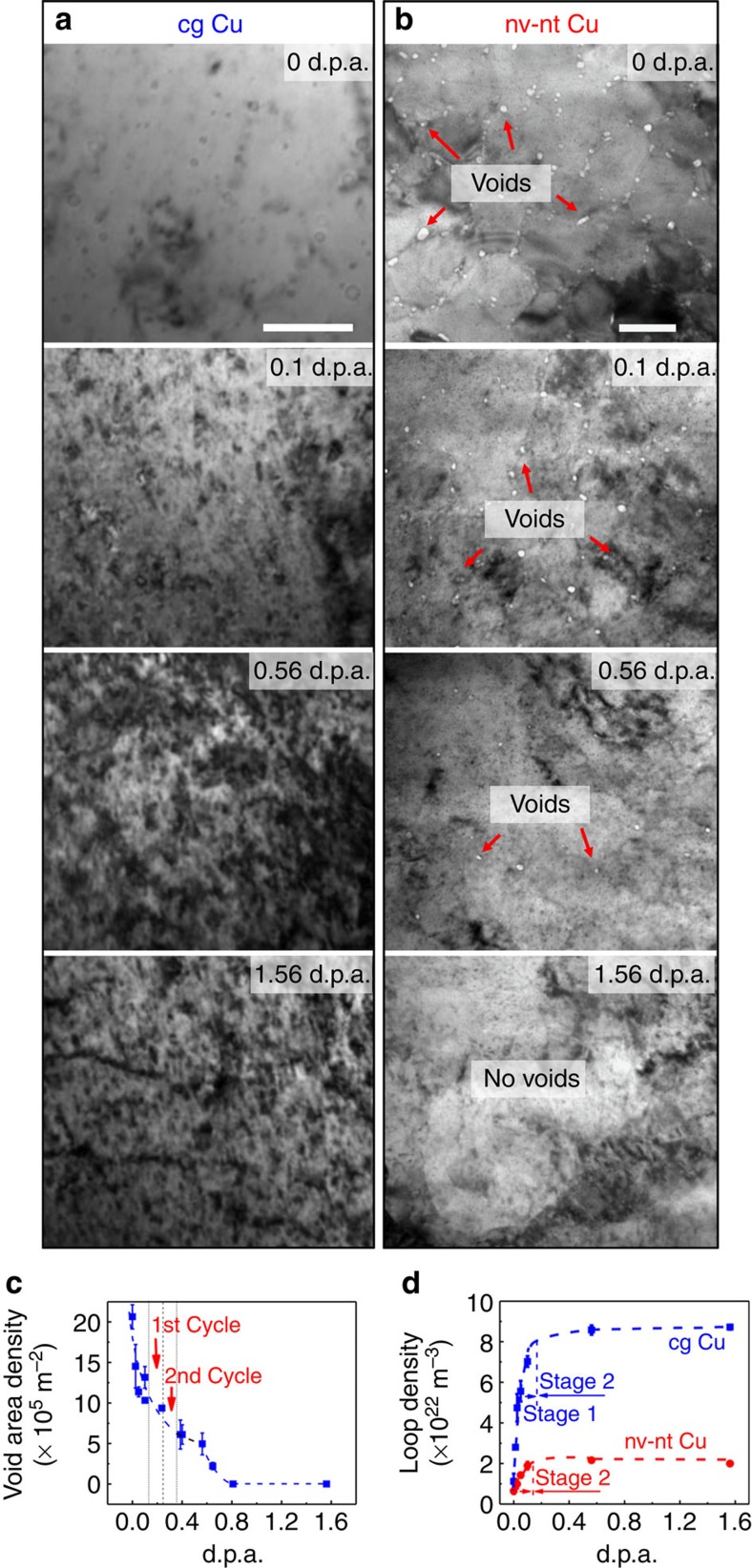
Superior radiation tolerance and void shrinkage in nv-nt Cu as evidenced by *in situ* Kr ion irradiation studies. TEM snapshots in (**a**) and (**b**) compare drastically different evolution of microstructure during *in situ* Kr ion irradiation of coarse grained (cg) and nv-nt Cu. (**a**) During initial radiation of cg Cu by 0.1 displacements per atom (d.p.a.), there is a rapid and prominent increase in density of defect clusters, the density of dislocation loops increased monotonically with dose and high-density dislocation segments were observed by 1.56 d.p.a.. Scale bar, 100 nm. (**b**) In contrast, in nv-nt Cu, the density of dislocation loops increased slightly with dose accompanied by a gradual elimination of nanovoids. Scale bar, 100 nm. (**c**) Up to 0.56 d.p.a., a significant decrease of area density of nanovoids was observed. By 1.56 d.p.a., nanovoids were mostly removed. The error bars of void density were determined from the s.d. from the average void density measured from several locations of the irradiated specimens. (**d**) A statistical study shows that the defect density in cg Cu increased rapidly to a much greater saturation level than that in nv-nt Cu.

Radiation response of nv-nt Cu was investigated via *in situ* Kr ion irradiation studies. TEM snapshots compare the drastic difference in evolution of microstructure during irradiation of coarse grained (cg) (Fig. 2a) and nv-nt Cu (Fig. 2b). During initial radiation of cg Cu by 0.1 displacements per atom (d.p.a.), there was a rapid, prominent increase in density of defect clusters; the density of dislocation loops increased monotonically with dose and a high density of dislocation segments was observed by 1.56 d.p.a. In contrast, in nv-nt Cu, the density of dislocation loops increased slightly with dose accompanied by a gradual elimination of nanovoids. By 0.56 d.p.a., a significant decrease of void density was observed. By 1.56 d.p.a., voids were mostly removed (Fig. 2c). However, nanotwins retained after radiation (Supplementary Fig. 2). A statistical study (Fig. 2d) shows that the defect density in cg Cu increased rapidly to a much greater saturation level than that in nv-nt Cu.

**Figure 3:**
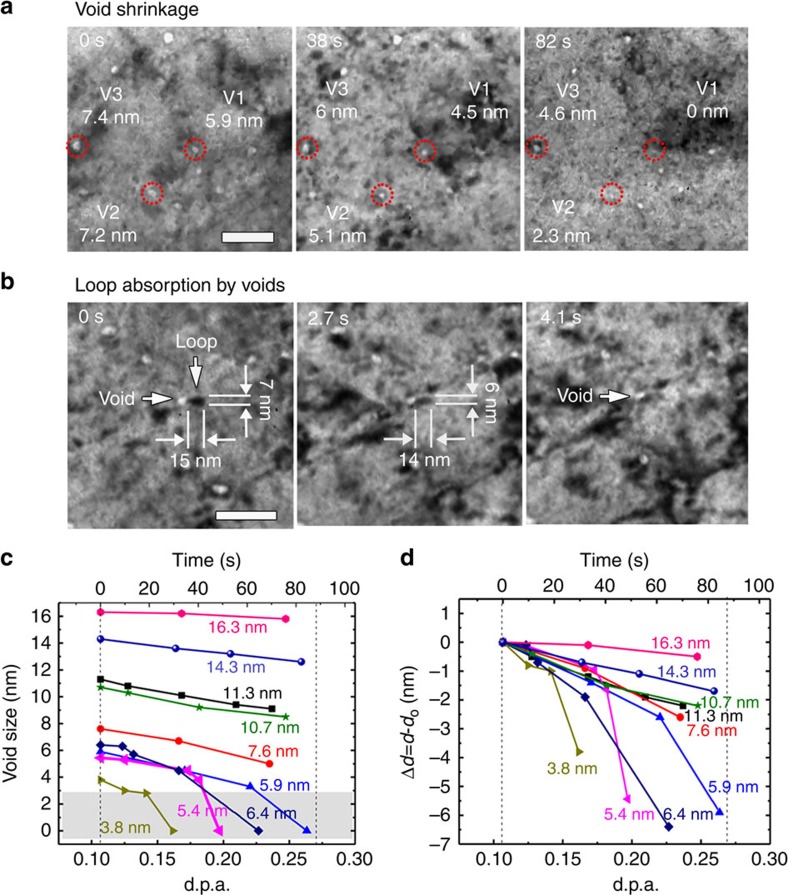
*In situ* Kr ion irradiation studies of nv-nt Cu unravelling continuous shrinkage of nanovoids and absorption of mobile dislocation loops by nanovoids. (**a**) *In situ* snapshots revealing shrinkage of numerous nanovoids over 0.11–0.26 d.p.a. At 0 s, 3 voids (V1-V3) with respective diameters of 5.9, 7.2 and 7.4 nm were tracked. At 82 s, V1 disappeared completely, while V2 and V3 decreased to 2.3 and 4.6 nm, respectively. Scale bar, 50 nm. (**b**) Sequential snapshots capturing the absorption of dislocation loops by voids over 0.13–0.14 d.p.a. (see Supplementary Movie 1 for detail). At 2.7 s, the loop was partially absorbed by the void. By 4.1 s, a complete absorption of the dislocation loop was observed. Scale bar, 50 nm. (**c**) Compiled chart showing the shrinkage of nanovoids with different diameters during *in situ* radiation. While larger voids shrank continuously during radiation, the rate of shrinkage is clearly greater for smaller nanovoids. When void diameter reduced to ∼3 nm (marked as grey band), there appeared to be an accelerated collapse of these tiny nanovoids, that is these voids vanished nearly instantaneously. (**d**) The evolution of reduction of void diameter Δ*d*=*d*−*d*_0_ with radiation dose and time, where *d*_0_ is the original void diameter.

Figure 2c shows that the void density declined sharply with increasing radiation dose, and by∼0.7 d.p.a., nanovoids were barely detectable. The average diameter of nanovoids in as-fabricated nv-nt Cu was∼10 nm with frequent appearance of nanovoids as large as 20 nm (Supplementary Fig. 3). Radiation led to substantial shrinkage of nanovoids, and by 0.65 d.p.a., most of nanovoids were below 10 nm in diameter. Figure 3a displays apparent shrinkage of nanovoids over 0.11–0.26 d.p.a. Three voids with initial diameters of 5.9 nm (marked as V1), 7.2 nm (V2) and 7.4 nm (V3) were chosen to illustrate the shrinkage process. These voids reduced diameters continuously. At 82 s, V1 disappeared completely while V2 and V3 contracted to 2.3 and 4.6 nm, respectively.

### Void–interstitial loop interaction

Absorption of interstitial loops by nanovoids was frequently observed. A typical absorption event captured by *in situ* TEM experiments is shown in Fig. 3b. At 0 s, an interstitial loop with dimension of 15 nm in length and 7 nm in width impinged a nanovoid, ∼6 nm in diameter. The loop shrank to 14 nm in length and 6 nm in width at 2.7 s, and was completely absorbed by the nanovoid by 4.1 s (see the Supplementary Movie 1). Figure 3c shows the variation of void size (**d**) with dose (time) for numerous nanovoids in nv-nt Cu. A majority of voids contracted continuously and gradually during radiation. When void diameter reduced to ∼3 nm, there appeared to be an accelerated contraction of these tiny voids. Figure 3d displays the reduction of void diameter, Δ*d*=*d*−*d*_0_, where *d*_0_ is the original diameter of voids. The greater shrinkage rate for the smaller voids than larger voids will be discussed later.

### Cyclic variation of mobile dislocation loop density in nv-nt Cu

**Figure 4:**
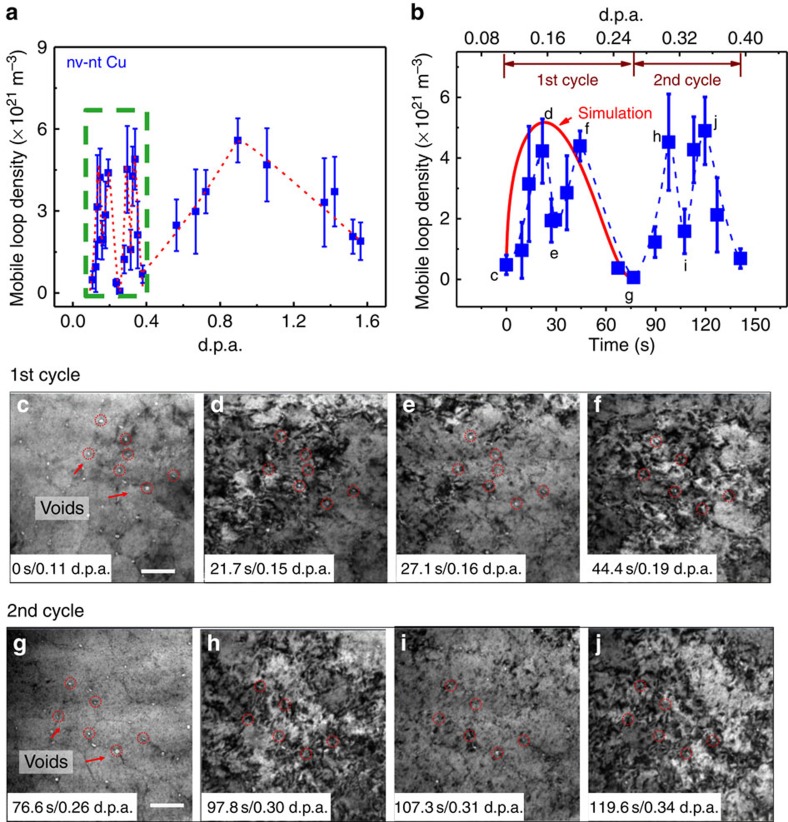
Significant cyclic variation of mobile loop density observed in nv-nt Cu subjected to *in situ* Kr ion irradiation within 0.4 d.p.a. (**a**) Statistic studies show cyclic variation of mobile dislocation loop density, with two short cycles during 0.1–0.4 d.p.a. (magnified in **b**), a 3rd much longer cycle, while little mobile dislocations were observed in cg Cu. In each of the first two cycles, two peaks and an intermediate valley were observed (**b**). The simulation of the 1st cycle is shown as a red solid line. TEM micrographs in **c**–**f** and **g**–**j** show cyclic variation of mobile dislocation loop density in two cycles (0.11–0.19 d.p.a.) and (0.26–0.34 d.p.a.). Scale bar, 100 nm in **c**; scale bar, 100 nm in **g**. (See Supplementary Movies 2 and 3 for more details on loop density variation for the 1st and 2nd cycle of radiation). The error bars for mobile loop density were determined from the s.d. from the average loop density measured from several locations of the irradiated specimens.

*In situ* radiation of nv-nt Cu also reveals an unusual phenomenon: cyclic variation of mobile dislocation loop density (Fig. 4a). Two short duration cycles were observed during radiation over 0.1–0.4 d.p.a., followed by a 3rd much longer cycle, whereas little mobile dislocations were observed in irradiated cg Cu. The first two prominent cycles had a similar period of ∼75 s (Fig. 4b), spanning across 0.11–0.34 d.p.a. Furthermore, each cycle contained two maxima and an intermediate local minima state. *In situ* TEM snapshots were captured to investigate the two repetitive cycles. In the first cycle, the density of mobile dislocation loops increased rapidly and reached a maximum by 21.7 s at 0.15 d.p.a. (Fig. 4d); it decreased to an intermediate level at 27.1 s (Fig. 4e); the population of dislocation loops then increased to a second maximum by 0.19 d.p.a. (Fig. 4f). This phenomenon repeated itself for a second cycle from 0.26 to 0.34 d.p.a. as shown in Fig. 4g–j (also see Supplementary Movies 2 and 3).

## Discussion

Void swelling is widely observed in irradiated face-centered cubic (fcc) metals^11^, in particular in austenitic stainless steels^16^,^17^,^48^, but the shrinkage of voids is rarely observed during radiation. The elimination of nanovoids in nv-nt Cu is thus unusual and must be related to the nv-nt microstructure.

**Figure 5:**
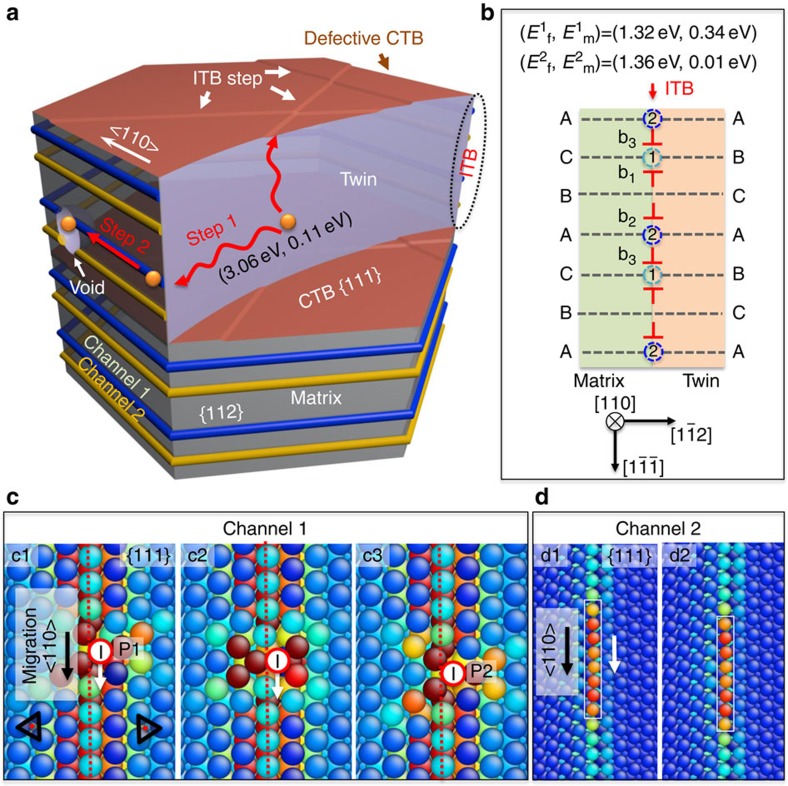
Absorption and diffusion of interstitials in nv-nt Cu. (**a**) Fast interstitial diffusion pipes enabled by ITB-CTB networks in nt Cu. (**b**) Two fast diffusion channels at ITBs and (**c**,**d**) the corresponding diffusion mechanisms. An interstitial created within the crystal will quickly migrate to ITBs or defective CTBs due to the low formation energy at these sites (labelled as step 1 in **a**). Once arrived at ITB-CTB networks, the interstitial can diffuse to a nanovoid at ITB via a rapid one-dimensional (1D) diffusion channel due to the low migration energy (step 2 in **a**), resulting in the shrinkage of the nanovoid. Topological model and atomistic simulations of ITB in an fcc structure, exploring that an ITB can be represented as an array of Shockley partial dislocations on each {111} plane as illustrated in the schematic (**b**), containing three repetitive partial dislocations (b_1_,b_2_ and b_3_). Two fast diffusion channels along <110> dislocation lines are identified as channel 1 and channel 2. The migration paths with lowest energy barriers along the two channels are calculated by the nudged elastic band (NEB) method as shown in (**c**) and (**d**), respectively. (**c**) For the channel 1, an interstitial initially stays at a dislocation core in an {111} layer sandwiched between b_1_ and b_3_. The interstitial then migrates downward to another low-energy site, with energy at the same level as its initial low-energy site. (**d**) For the channel 2, an interstitial has a spreading core associated with the distributed free volume along <110> dislocation line. The migration of the distributed interstitial requires a very low energy barrier (0.01 eV) displaying a crowdion-type of behaviour.

TB networks enable absorption and rapid transportation of defects/defect clusters for the following reasons. First, TBs are effective defect sinks. By using molecular statics simulations, we compared the formation and migration energies (*E*_f_, *E*_m_) of interstitials inside crystal and at ITB-CTB network (Fig. 5a). The formation energy for an interstitial within the crystal lattice is very large,∼3 eV, compared with 1–2 eV on CTBs and ITBs (Fig. 5b and Supplementary Fig. 4), implying interstitials prefer to stay at TBs. Furthermore interstitials in grain interior have very low migration energy (∼0.11 eV, see detailed diffusion mechanism in Supplementary Fig. 5), permitting their rapid migration to nearby CTBs or ITBs (marked as step 1 in Fig. 5a). Second, once arrived on ITB-CTB networks, defects or their clusters can be transported rapidly (similar to 1D diffusion) along fast diffusion channels to nanovoids (marked as step 2 in Fig. 5a). For regular ITBs consisting of sets of three adjacent Shockley partials^49^, we characterized two fast diffusion paths along dislocation lines that experience the kinetic barrier of 0.34 eV for channel 1 at tensile sites sandwiched by two partial dislocations (b_1_ and b_3_), and 0.01 eV for expeditious 1D crowdion diffusion in channel 2 (Fig. 5b), and the detailed diffusion mechanisms are displayed in Fig. 5c,d, respectively. A complete view of the interstitial migration at ITB-CTB network is provided in Supplementary Fig. 4, which, for example, shows the kinetic energy barrier is as low as 0.01–0.16 eV for channels along ITB-CTB junctions in nt Cu. Third, although CTBs have low mobility, the capture of dislocation loops by CTBs can create abundant minuscule ITB steps^50^,^51^,^52^,^53^, which contain groups of highly mobile Shockley partials^42^. *In situ* nanoindentation studies have shown that ITB steps in nt Cu can migrate rapidly during deformation at low stress^54^. Hence, the 3D ITB-CTB networks can transport defects and their clusters efficiently to nanovoids. Further discussions of fast diffusion channels on ITB-CTB network in nt Cu is provided in Supplementary Note 1.

Forgoing discussion suggests that a large number of loops will be transported to voids via ITB-CTB networks, whereby they are annihilated. Here we examine loop-void interaction mechanisms in detail. First, void size plays an important role in capturing and storing defects in nv-nt Cu. The analytical anisotropic solution of stress state between two voids calculated by using complex variable method (see Supplementary Fig. 6) indicates the existence of significant tensile stress surrounding voids. Smaller voids generate higher stress field near void surfaces, while larger voids introduce higher stress over a longer range. When a Frank loop approaches a nanovoid, the loop migration rate increases significantly as tensile stress induces a substantial reduction of formation and migration energies of the loop (detailed calculations by molecular statics simulations are provided in Supplementary Fig. 7).

**Figure 6:**
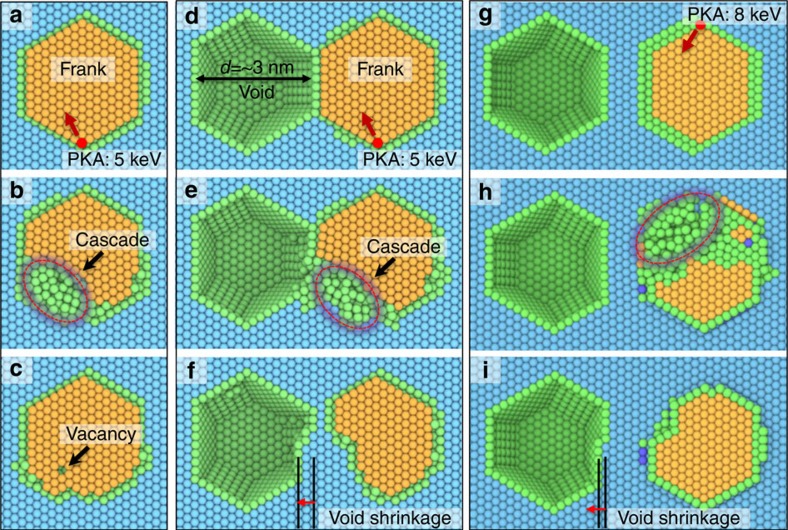
Two-dimensional projected view of interstitial loop–nanovoid interactions. (**a**) For a stand-alone Frank loop, a 5 keV primary knock-on atom (PKA) generates a cascade at one corner of the loop (**b**). During the quenching process, the cascade shrinks, accompanied by the recovery of the Frank loop. After the retreat of the cascade, the Frank loop evolves back to its original configuration, except a vacancy at the loop and an interstitial out of the loop (a Frenkel pair) (**c**). (**d**) For a Frank loop immediately next to a void (*d*=3 nm), a similar cascade is performed. (**e**) Accompanying the retreat of the cascade, the interstitials are absorbed into the void (**f**), leading to a shrinkage of the void and substantial removal of the Frank loop. No defects appear out of the Frank loop. (**g**) For a Frank loop ∼1 nm away from a void (*d*=3 nm), a similar cascade generated by an 8 keV PKA is performed (**h**). The interstitials of the Frank loop are attracted into the void (**i**), leading to shrinkage of the void and Frank loop. No defects appear out of the Frank loop in cases **d** and **h**. Detailed processes are provided in Supplementary Movies 4, 5, 6.

Second, MD simulation reveals dynamic process through which a void absorbs a neighbouring dislocation loop. Three scenarios subjected to self-ion irradiations, were compared, including a stand-alone Frank (interstitial) loop, a pair of nanovoid and Frank loop in immediate contact, and the similar pair that are separated by∼1 nm as shown in Fig. 6. As shown in Supplementary Movie 4, during irradiation, the individual Frank loop was disturbed, but only slightly changed its shape after a cascade (Fig. 6a–c). In parallel, the Frank loops immediately contacting the void (Fig. 6d–f) or slightly separated from the void (Fig. 6g–i) were prominently absorbed by the void after radiation (Supplementary Movies 5 and 6). The amount of net interstitials (inside a Frank loop) absorbed by a void depends on the energy and fluence of primary knock-on atoms. Supplementary Fig. 8 and Supplementary Movie 7 show one of the cases wherein multiple cascades (3 incident ions) led to substantial absorption of a Frank loop and shrinkage of the void, leaving behind stacking faults, vacancies and a prismatic loop.

*In situ* radiation studies show that cyclic variation in density of mobile dislocations is directly related to void shrinkage in nv-nt Cu. During radiation, the interaction of radiation-induced defects and TBs provides a continuous source for mobile interstitial loops. In contrast, nanovoids are sinks for these defects. The net accumulation of mobile loops is thus a competition between these two processes. The simulation developed from such concept (see Supplementary Figs 9 and 10 for details) yields time-dependent evolution of mobile loop density (shown by solid red curve in Fig. 4b), in qualitative agreement with experimental observations. Further discussion of cyclic variation of mobile dislocation loop density is provided in Supplementary Note 1.

Foregoing discussions highlight several uniqueness of nv-nt architecture in achieving unprecedented radiation tolerance. First, compared with high-angle grain boundaries in nanocrystalline metals, TBs are comparable defect sinks, but much more stable under radiation. Second, nt metals have extraordinary 3D ITB-CTB networks with rapid diffusion channels that act as highways to rapidly transport point defects and their clusters. The fast diffusion channels discovered in nt metals have one dimensional nature and are much more efficient than high-angle grain boundaries to transport defect clusters to desirable destination (nanovoids). Third, abundant nanovoids at the end of these highways have capacity to store and eliminate these defect clusters. In the absence of nanovoids, even if TB networks remain actively transporting defects, there is no space to eliminate defect clusters in a timely manner and hence defect density will build up gradually. For comparison, we fabricated cg Cu with nanovoids with dimensions of ∼100 × 200 nm, which were the smallest achievable dimension by using the focused ion beam technique at the time of experiment. *In situ* radiation experiments on these cg Cu films show no shrinkage of nanovoids after radiation to a dose of 1.56 d.p.a., implying that the removal of nanovoids is a unique phenomenon in nv-nt Cu films (Supplementary Fig. 11).

In summary, we report on a new method beyond existing approaches^55^ to achieve extraordinary radiation tolerance by using nv-nt architecture. The self-healing effect observed in this study shall not be interpreted as mitigation of void swelling only. Instead the proper insertion of nanovoids (along ITBs) ensures that mobile defect clusters can be ‘stored', and consequently permits continuous and expeditious removal of mobile dislocation loops. The ITB-CTB networks in nt metals enable rapid transportation of radiation-induced defect clusters to nanovoids and thus lead to their mutual recombination. The concept developed from this study –combination of nanovoids with nanotwin networks– not only helps us to understand defect mitigation mechanisms in irradiated materials, and but may also stimulate the design of damage tolerant materials in general that are subjected to other extreme environments, such as high stress and high pressure impact.

## Methods

### Specimen preparation

Fully dense cg Cu foil with thickness of ∼20 μm and nanotwinned Cu films with nanovoids with thickness of 1.5 μm were prepared through magnetron sputtering by using 99.995% purity Cu target on HF-etched Si(110) substrates. Subsequent annealing of free-standing cg Cu foil at 300 °C for 1 h was performed to obtain large grain size. Before deposition, the chamber was evacuated to a base pressure of ∼5 × 10^−8 ^torr. TEM samples were prepared by dimpling, low-energy (3.5 keV) Ar ion milling and subsequent ion polishing.

### *In situ* Kr ion irradiation

*In situ* Kr^++^ ion irradiation at 1 MeV was performed for cg and nv-nt Cu at room temperature in the intermediate voltage electron microscope (IVEM) at Argonne National Laboratory, where an ion accelerator was attached to a HITACHI H-9000NAR microscope. The microscope was operated at 200 kV and kept on during radiation to record the microstructural evolution. The average dose rate was 1.8 × 10^−3 ^d.p.a. s^−1^. A CCD camera was used to capture microstructural evolution during radiation at 15 frame s^−1^. Stopping power and range of ions in matter simulation was used to estimate the displacement damage profile and Kr^++^ ion distribution^56^. The results (see Supplementary Fig. 12) show that most of the Kr^++^ ions at 1 MeV will penetrate through TEM foils, which are ∼100 nm in thickness, measured by using the Kossel–Mollenstedt fringes captured under two-beam conditions.

### Molecular statics/dynamics simulations

Molecular statics with the nudged elastic band method^57^ was used to calculate the formation/migration energy (*E*_f_/*E*_m_) for interstitials at TBs and in the bulk, with the embedded atom method interatomic potentials for Cu^58^. Twinned structures (Supplementary Fig. 13) were created by successively gliding Shockley partial dislocations on each (111) plane in a single crystal^49^,^59^. We introduced interstitials, one at a time, at all possible sites in the twinned structure and subsequently calculated the corresponding *E*_f_ and *E*_m_ with respect to migration path.

Molecular dynamics was used to study the void–Frank loop interaction under cascades. The box size is ∼200 × 100 × 100 Å. A void was created in a shape of Wulff construction with a radius of ∼1.5 nm. Frank loop takes a hexagonal shape with six <110> sides and radius of ∼1.5 nm. Embedded atom method interatomic potential developed by Mishin *et al*.^60^ was used to describe the interatomic interaction, splined to the ZBL repulsive potential for interatomic distances <0.5 Å. MD models were initially relaxed at 300 K. Cascade simulations were performed along different directions for primary knock-on atoms (PKA) with kinetic energy 5–8 keV. MD simulation stopped till the cascade cooled down without obvious rearrangement of atoms.

## Author contributions

Y.C. performed *in situ* radiation and microscopy experiments under the supervision of H.W. and M.A.K. Y.C., K.Y.Y. and Y. L. performed sample fabrication by magnetic sputtering. Theoretical simulations were constructed by Y.C. and S.S., supervised by J.W. X.Z. supervised the entire project. All authors commented on the manuscript.

## Additional information

**How to cite this article:** Chen, Y. *et al*. Damage-tolerant nanotwinned metals with nanovoids under radiation environments. *Nat. Commun*. 6:7036 doi: 10.1038/ncomms8036 (2015).

## Supplementary Material

Supplementary Information Supplementary Figures 1-13, Supplementary Notes 1-2 and Supplementary References.

Supplementary Movie 1 (Corresponding to Fig. 3b in the text) *In situ* Kr ion irradiation studies of nv-nt Cu demonstrating absorption of mobile dislocation loops by nanovoids over 0.13-0.14 dpa. The area in the box contains the point of interest. At 2.7 s, the loop was partially absorbed by the void. By 4.1 s a complete absorption of the dislocation loop was observed

Supplementary Movie 2 (Corresponding to Fig. 4c-f) *In situ* Kr ion irradiation demonstrating 1st cyclic variation of mobile loop density observed in nv-nt Cu subjected to *in situ* Kr ion irradiation over 0.11-0.19 dpa. In the 1st cycle, two peaks and an intermediate valley of mobile dislocation density were observed. The playing speed of this movie is accelerated 3 times.

Supplementary Movie 3 (Corresponding to Fig. 4g-j) *In situ* Kr ion irradiation demonstrating the 2nd cyclic variation of mobile loop density observed in nv-nt Cu subjected to *in situ* Kr ion irradiation over 0.26-0.34 dpa. The phenomenon in this cycle repeated that seen in the 1st cycle. Similar to the 1st cycle, in the 2nd cycle, two peaks and an intermediate valley of mobile dislocation density were observed. The playing speed of this movie is accelerated 3 times.

Supplementary Movie 4 (Corresponding to Fig. 6a-c) MD simulation showing the structural stability of a Frank loop under cascade by a 5 keV primary knock-on atom (PKA). For a standalone Frank loop (without voids nearby), the PKA generates a cascade in one corner of the loop. During the quenching process, the cascade shrinks, accompanied by the recovery of the Frank loop. After the retreat of the cascade, the Frank loop evolves back to its original configuration, except a vacancy at the loop and an interstitial out of the loop (A Frenkel pair).

Supplementary Movie 5 (Corresponding to Fig. 6d-f) MD simulation showing the absorption of a Frank loop next to a void. For a Frank loop next to a void (d = 3 nm), a cascade is generated by a 5 keV PKA (same condition as that in Supplementary Movie 4). Accompanying by the retreat of the cascade, the interstitials are attracted into the void, leading to a shrinkage of the void and Frank loop. No defects appear out of the Frank loop.

Supplementary Movie 6 (Corresponding to Fig. 6g-i) MD simulation showing the absorption of a Frank loop 1 nm away from a void. For a Frank loop ~ 1 nm away from a void (d = 3 nm), a similar cascade generated by a 8keV PKA is performed. The interstitials of the Frank loop are attracted into the void, leading to a shrinkage of the void and Frank loop. No defects appear out of the Frank loop.

Supplementary Movie 7 (Corresponding to Supplementary Figure 9) MD simulations of absorption of a Frank loop by a void under cascade by three 5 keV PKAs. 3D view shows a cascade occurred over most of the loop and the void shank with the absorption of interstitials in the Frank loop. The Frank loop was destroyed by the cascade, generating other defects such as stacking faults, vacancies, and a prismatic loop.
