# Supplementary material for: Damage-tolerant nanotwinned metals with nanovoids under radiation environments
Source: Nat Commun. 2015 Apr 24;6:7036. doi: 10.1038/ncomms8036 (PMC4421808; doi:10.1038/ncomms8036)
Supplement: Supplementary Information — Supplementary Figures 1-13, Supplementary Notes 1-2 and Supplementary References. [file ncomms8036-s1.pdf]

Supplementary Figures

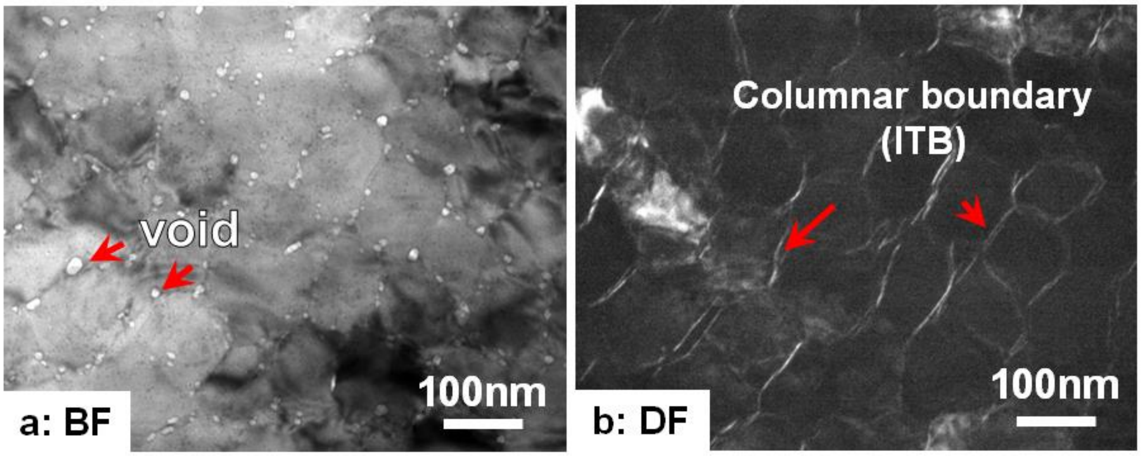

Supplementary Figure 1. (a) Plan-view transmission electron microscopy (TEM) micrograph showing the as-prepared nv-nt Cu film containing abundant nanovoids primarily surrounding columnar domain boundaries. (b) Dark-field image at the same area showing clear columnar domain boundaries.

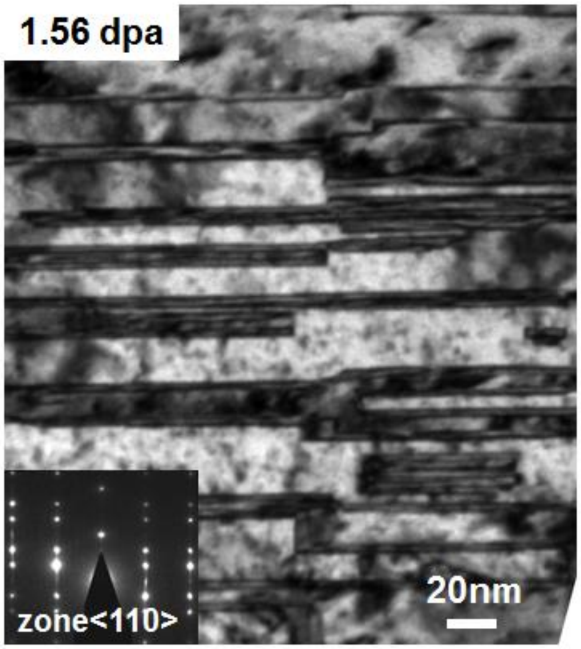

Supplementary Figure 2. Cross-section TEM micrograph of irradiated nt Cu up to 1.56 dpa showing the remarkable retention of nanotwins after irradiation. The average twin thickness remained ~ 15 nm. The inset of SAD pattern confirms the retention of nt structure in the irradiated nt Cu.

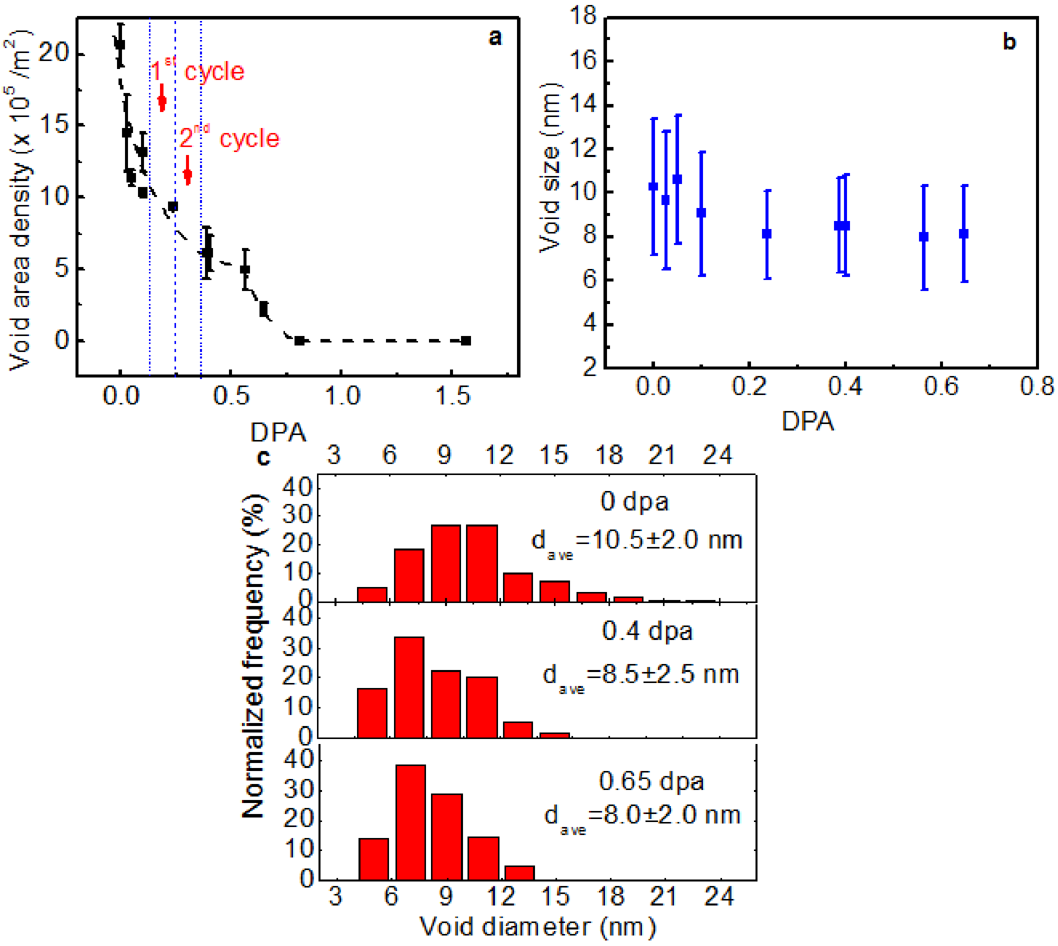

Supplementary Figure 3. Evolution of void density and size during radiation. (a) A statistical study displays that the void density decreases sharply with increasing radiation dose. By ~ 0.7 dpa, the voids were barely detectable. (b) With increasing irradiation dose, the average diameter of voids decreases from ~ 10 to 8 nm. (c) The statistical distribution of void diameters before (0 dpa) and after radiation (0.4 and 0.65 dpa) shows contraction of large voids.

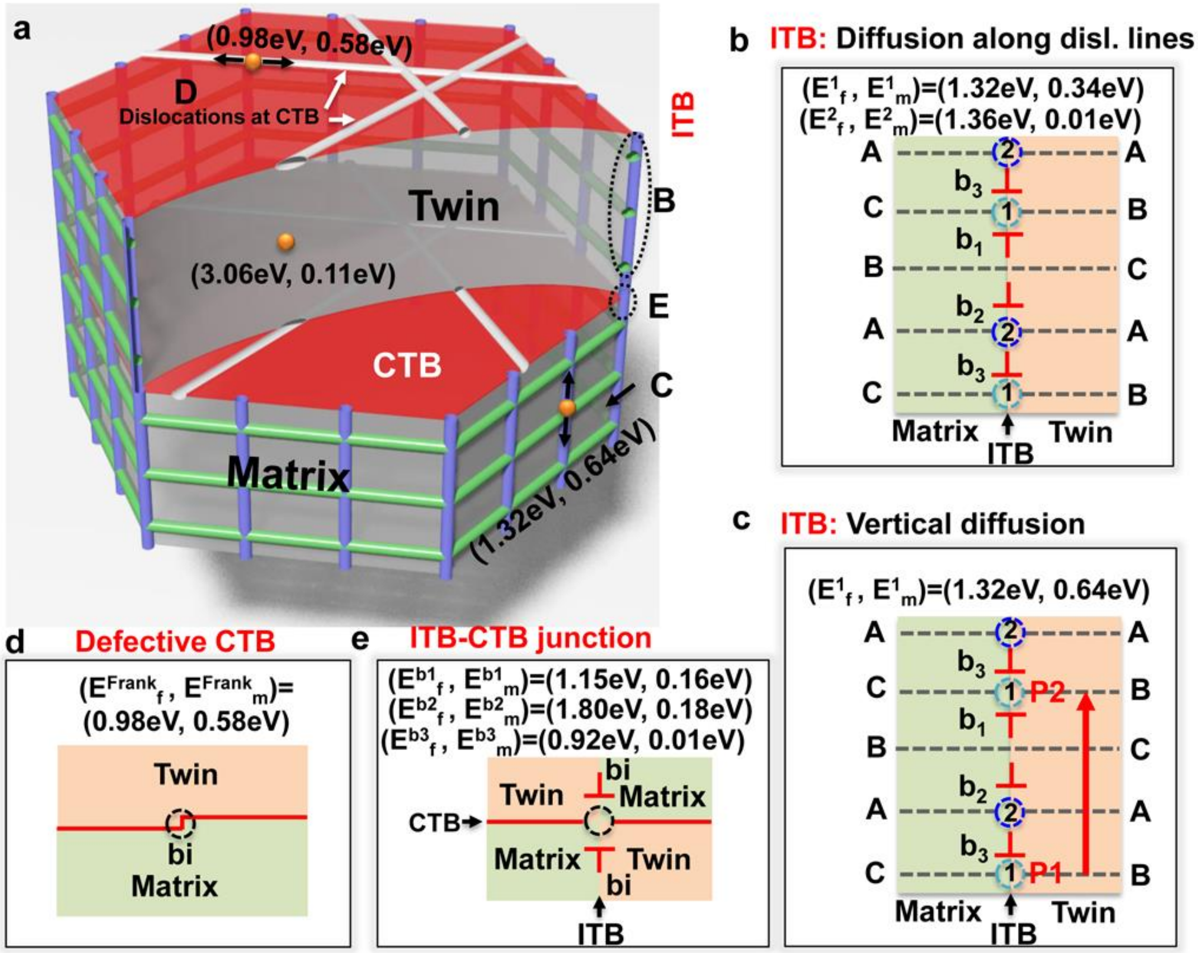

Supplementary Figure 4. (a) Fast interstitial diffusion pipe networks in nt Cu. (b-e) The formation and migration energies of interstitials at ITBs, CTBs and ITB-CTB junctions are provided. The detailed calculation in crystal and at ITBs is displayed in Supplementary Figure 5 to exemplify the calculation method. In general, the formation energy at ITB-CTB network (1~2eV) is lower than that in crystal (3eV). When an interstitial is created within the crystal, the difference in the formation energy drives the interstitial to the 1D fast diffusion pipes, either at ITBs or defective CTBs.

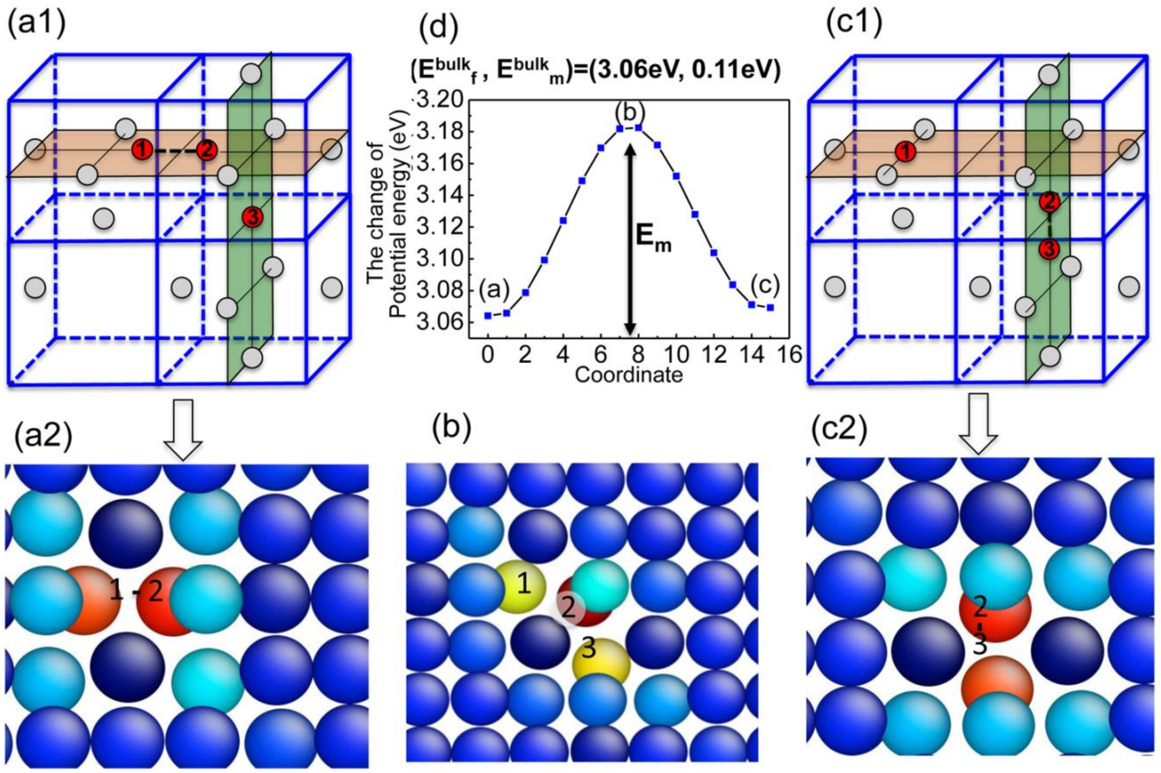

Supplementary Figure 5. (a1-a2) Radiation induced interstitial stays as a split <001> dumbbell configuration with the lowest formation energy configuration (3.06eV). The migration of the interstitial is coordinated by rotating the dumbbell configuration by 90° with a translation of the center of mass by one nearest-neighbor distance (b, c1-c2), overcoming a tiny kinetic energy barrier (0.11eV) (d).

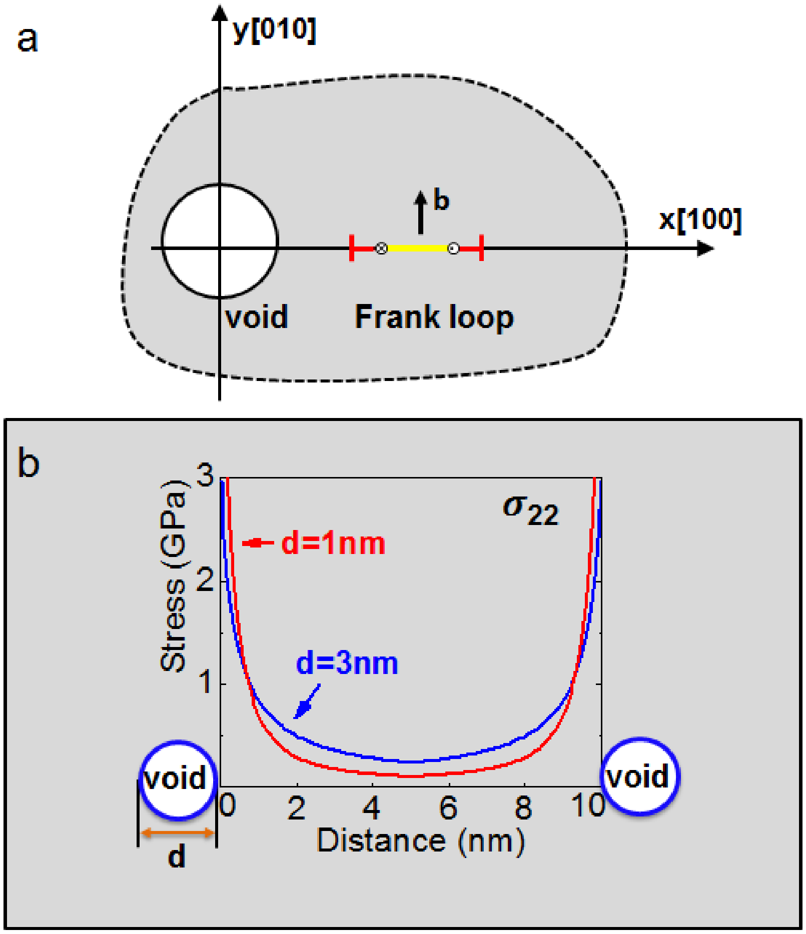

Supplementary Figure 6. (a) The interaction between a loop and a void with the stress field [$\boldsymbol{\sigma}$]. We choose one type of loops (Frank loop) here to demonstrate the attraction. With the known Burgers (**b**= [010]) and dislocation line ($\xi = [0\ 0\ \bar{1}]$), we can calculate the Peach-Koehler force as [$-\sigma_{22}$, σ_{21}, 0] by ($\mathbf{b}\cdot\boldsymbol{\sigma}$) × ξ. Thus, σ_{22} plays a key role in attracting the loop towards the void in this case. (b) The analytical calculation of tensile stress state (σ_{22}) between two voids by using complex variable method (*1*) indicates that smaller voids generate higher stress near the void surfaces, while larger voids introduce higher stress over a longer range. The stress level at surface is estimated by $P = 2\gamma / R$, where R, γ are the radius of voids and surface energy of void surface (~ 1.4 J/m^2 for Cu (*2*)).

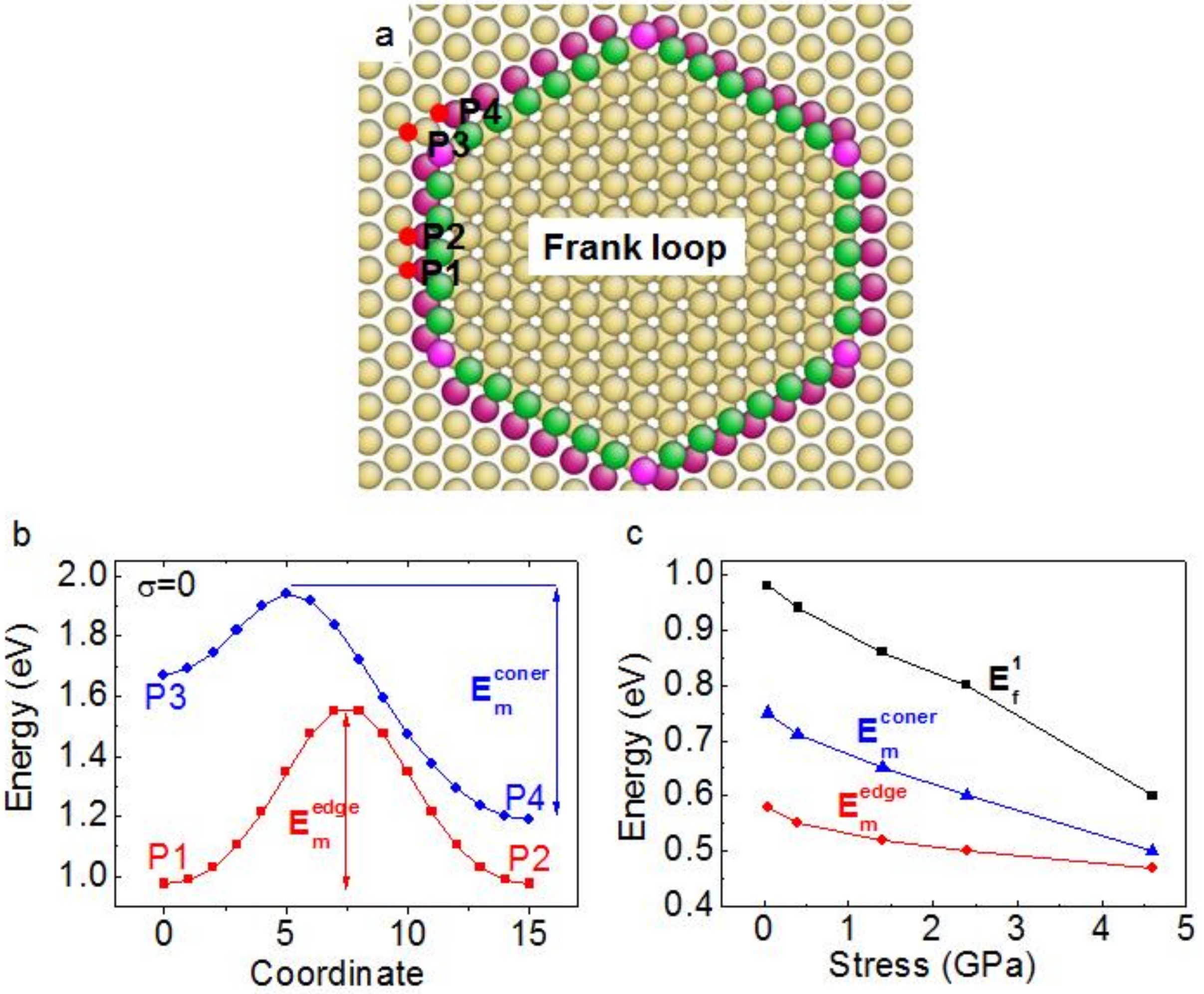

Supplementary Figure 7. Interstitial formation and migration energies (E_f, E_m) along a Frank loop derived by molecular statics calculations. (a) Atomistic structure of a Frank loop. (b) The interstitial formation energy at different sites along the loop line under zero applied stress. The migration paths with lowest energy barriers at loop side and corner are provided by NEB method. (c) The interstitial formation and migration energies at different sites along the loop line under applied stress normal to Frank loop. With the increase of applied tensile stress, the interstitial formation and migration energies are significantly reduced.

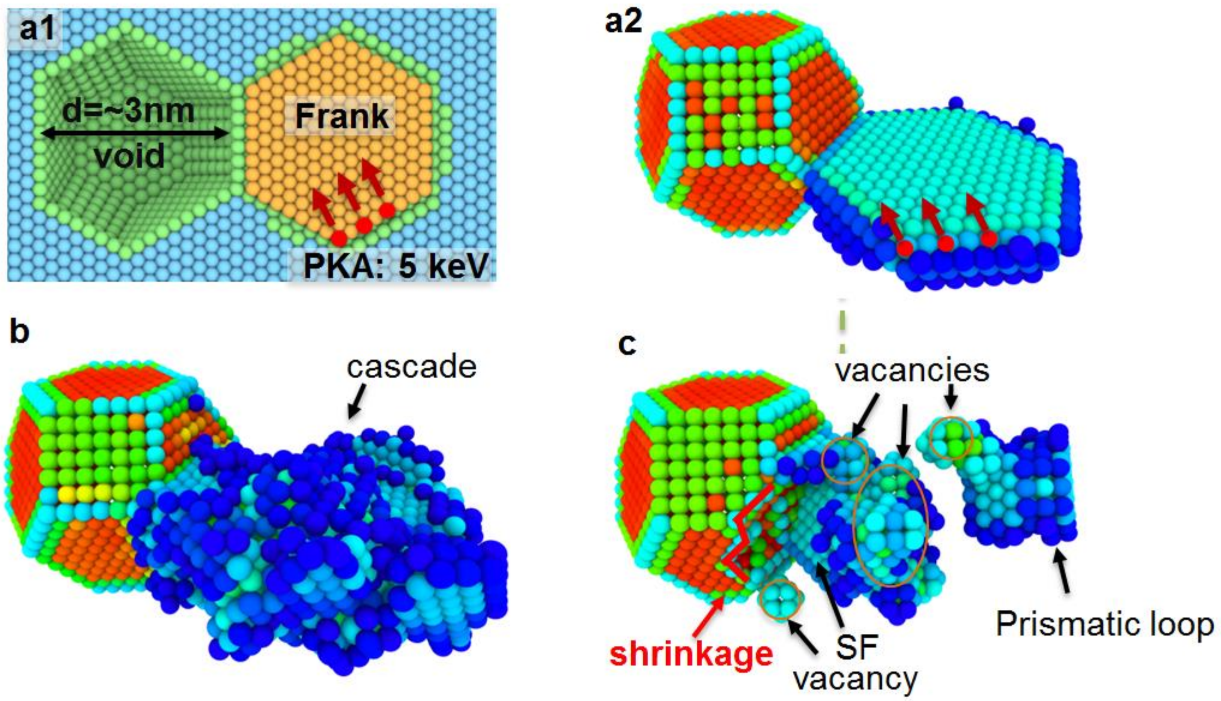

Supplementary Figure 8. MD simulations of absorption of a Frank loop by a void under cascade. (a1-a2) 2D and 3D views of a Frank loop close to a void. (b) a snapshot of cascade structure when the Frank loop was bombarded by 5 keV primary knock-on atoms (PKA). (c) A cascade occurred over most of the loop and the void shank with the absorption of interstitials in the Frank loop. The Frank loop was destroyed by the cascade, generating other defects such as stacking faults, vacancies, and a prismatic loop (For details, see Supplementary Movie 7).

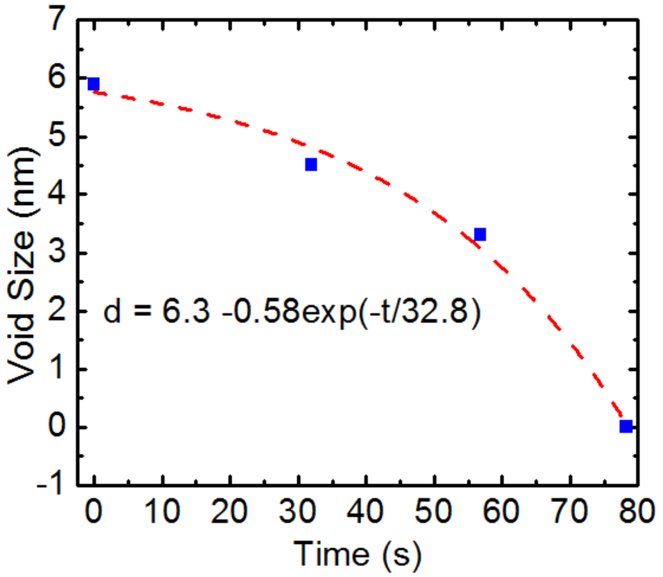

Supplementary Figure 9. The evolution of void size with time for a typical nanovoid with initial diameter of ~ 6 nm. The time dependent reduction of void diameter is fitted as the red dash line using inserted analytical formula.

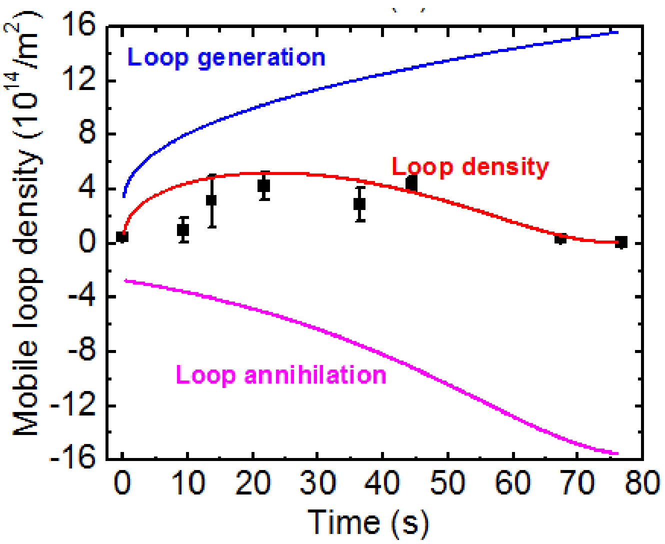

Supplementary Figure 10. The evolution of dislocation density with time. Simulated evolution curve of dislocation density (red) is a summation of dislocation annihilation (pink) and dislocation generation (blue) curves. Please note that the pink line (due to loop induced annihilation of loops) was derived from empirical fitting of loop diameter (Supplementary Figure 9). The blue line was obtained by using $\rho_{\mathbf{gen}}(t) = a + bt^n$ (equation 2 in the Supplementary Note 2), where n = 0.43 from literature. Two fitting parameters, a and b, were thus used to obtain the red solid line to fit the experimentally determined time dependent variation of loop density (solid square data points).

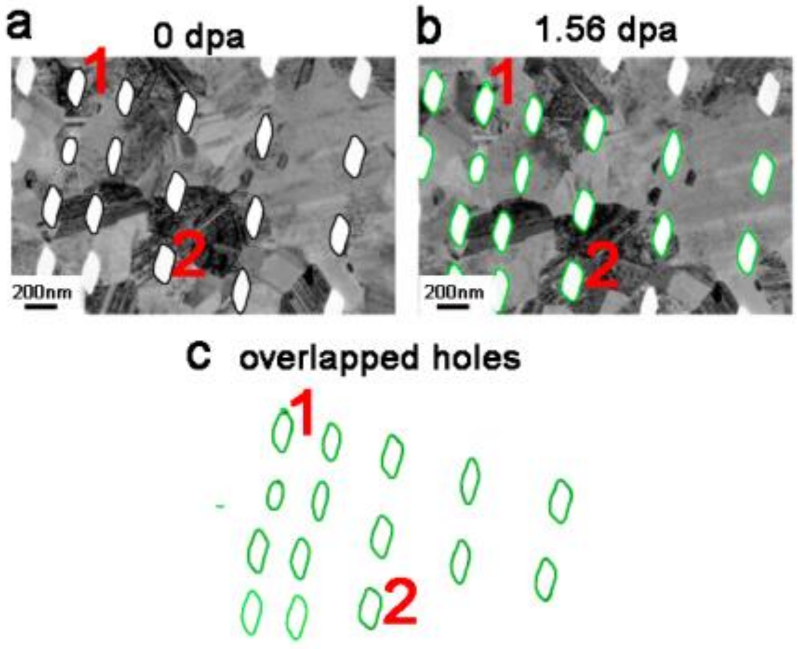

Supplementary Figure 11. The stability of nanovoids fabricated by the focused ion beam (FIB) technique in cg Cu subject to *in situ* Kr ion irradiation up to a dose of 1.56 dpa. (a) Before radiation, nanovoids with diameter of 100 – 200 nm were drilled by FIB technique. (b) After irradiation to 1.56 dpa, there is no detectable change in shape of the nanovoids. (c) Overlapping of nanovoids before and after irradiation confirmed negligible change in shape and size of nanovoids under radiation. Two typical nanovoids (labeled as 1 and 2) were tracked to examine the evolution of voids.

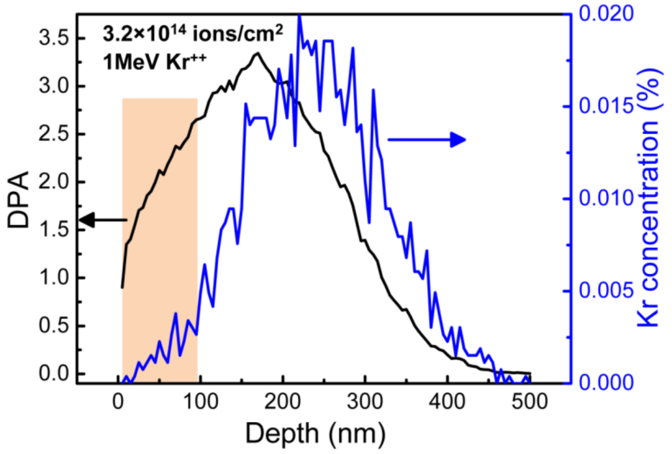

Supplementary Figure 12. SRIM simulation showing the dpa and Kr concentration profiles along the ion penetration depth for 1 MeV Kr ions. The first 100 nm thick TEM foil was subjected to an average dose of ~ 1.56 dpa and most Kr ions penetrated through the TEM foil, leaving behind radiation damage and insignificant Kr concentration.

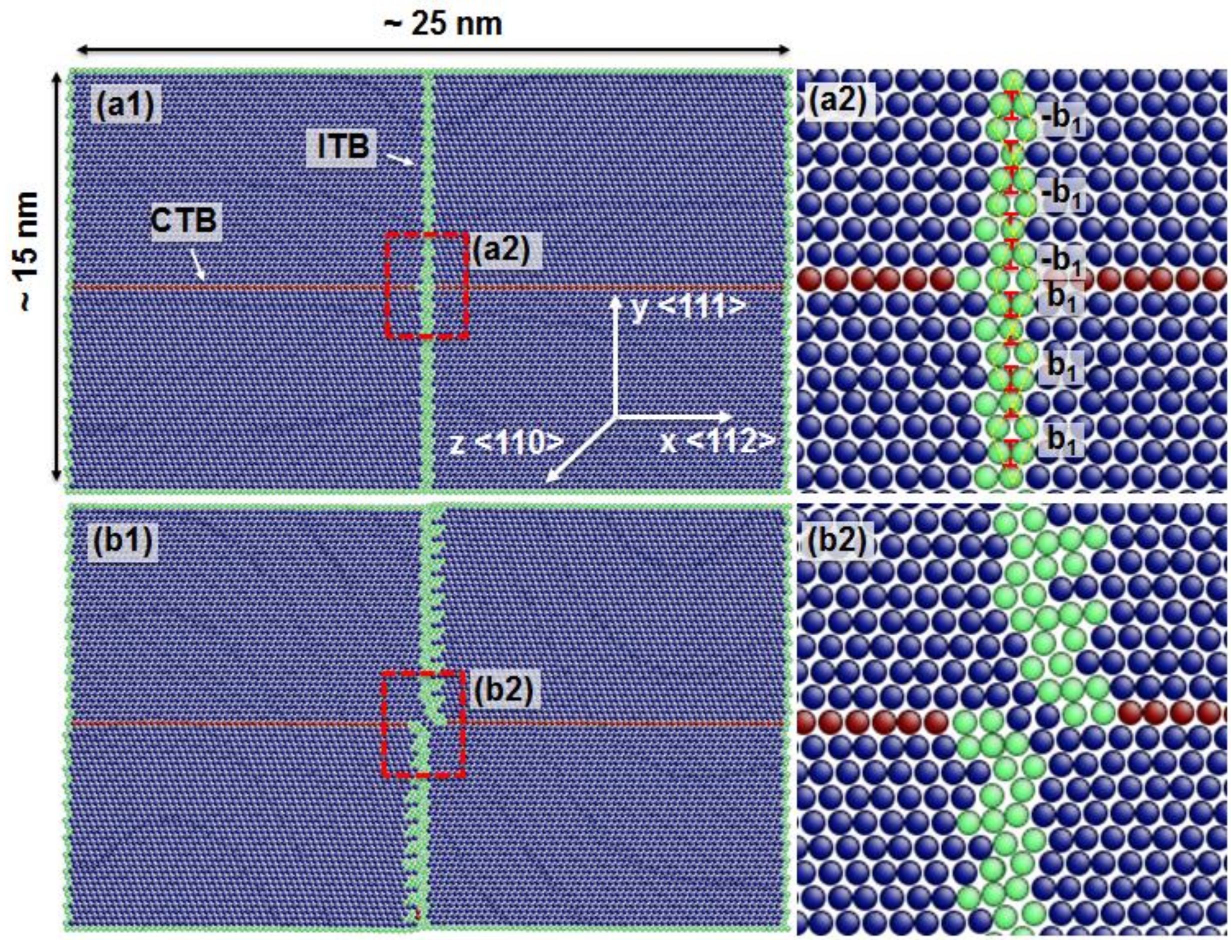

Supplementary Figure 13. Simulation models with respect to one type of ITB-CTB junction (b_1/b_1) before relaxation. The box size is ~ 25, 15, 3 nm in x, y and z direction. The nt structure has a rigid boundary (~ 10 Å thickness) in x and y direction. Periodic boundary condition was applied in the z direction. (A2) shows a Shockley partial dislocation on each (111) plane (only b_1 is marked) with a repetitive sequence (b1, b2 and b3) in every 3 layers (*3*). (B1-B2) show the relaxed structure of the junction from (A1) and (A2).

Supplementary Note 1: Fast diffusion channels associated with ITB-CTB network in nt Cu

Nt Cu intrinsically contains an ITB-CTB network that can act as fast diffusion channels for point defects. A systematic calculation of formation and migration energies (E_f, E_m) for

interstitials inside crystal and along various types of diffusion pipes (see Supplementary Figure 4) is performed using atomistic simulation to obtain a complete view of diffusion channels in nt Cu.

Inside the crystal, radiation induced interstitials stay as a split <001> dumbbell configuration with the lowest formation energy configuration (3.06eV) (Supplementary Figure 5). The migration of the interstitial is coordinated by rotating the dumbbell configuration by 90° with a translation of the center of mass by one nearest-neighbor distance, with a kinetic energy barrier of 0.11eV, which indicates that interstitials inside Cu crystal can quickly migrate onto twin boundaries nearby. The migration mechanism in the bulk we characterized in this study is identical to what suggested by Sørensen *et al.*(*4*) and Zhao and Shimomura (*5*).

At defective CTBs, radiation introduces ITB steps consisting of two types of dislocations: Shockley and Frank partials, generated either by dislocation-twin boundary interactions or accumulation of interstitials at CTBs. The dislocation-CTB interactions have been intensively discussed and summarized in literature (*6*-*9*). These interactions result in mobile Shockley partials or immobile Frank partials on the CTB. On the other hand, the difference in formation energy and low migration energy drive interstitials generated in the bulk to accumulate at defective CTBs. As <001> dumbbell configuration cannot form on (111) twinning planes, interstitials could stay at CTBs without significant migration. Therefore, accumulation of interstitials leads to the formation of dislocations/dislocation loops at CTBs, e.g. Frank loops. It is worth mentioning that Shockley partials at twinning planes can quickly migrate on CTBs and deliver interstitials together associated with their glide on CTBs while immobile Frank partials can provide fast diffusion channels (see Supplementary Figure 4).

ITB structure can be represented as an array of Shockley partial dislocations on each {111} plane (*3*, *10*), as illustrated in the schematic (Supplementary Figure 4), containing three

repetitive partial dislocations (b_1, b_2 and b_3). Two fast diffusion channels along <110> dislocation lines are marked as channel 1 and channel 2. For channel 1, an interstitial initially stays at dislocation core in {111} layer sandwiched by b_1 and b_2. With the coordination of atoms around, the interstitial migrates downward to another low-energy site, same as initial low-energy site. For channel 2, an interstitial has a spreading core associated with the distributed free volume along <110> dislocation line, and migrates with a super low energy barrier (0.01eV) by crowdion-like behavior.

The amount of ITB-CTB junction lines becomes significant in nt structure with high-density TBs. We demonstrate three different structures with various combinations of partial dislocation at ITB-CTB junctions (b_1/b_1, b_2/b_2 and b_3/b_3) and study the formation and migration energies of interstitials at and along these junctions. In general, all these three channels have low formation and migration energies for interstitials (Supplementary Figure 4), among which the diffusion channel with the combination of b_3/b_3 shows a crowdion-like diffusion with an ultra-low kinetic energy barrier of 0.01eV.

Supplementary Note 2: Discussion of cyclic variation of mobile dislocation loop density

We quantitatively explain this cyclic variation phenomenon as follows. The time (dose) dependent reduction in density of mobile interstitial loops, $\rho_{\text{anni}}(t)$, due to their annihilation at voids is given by

$$\rho_{\text{anni}}(t) = \frac{\Delta V}{d_L^2 \times b} \qquad (1)$$

where ΔV is the reduction in volume of voids due to absorption of dislocation loops (or segment), which can be estimated by simulating the time dependent evolution of void diameter and void

density (Supplementary Figure 9). Meanwhile the production of density of mobile loops, $\rho_{\mathbf{gen}}(t)$ can be estimated as

$$\rho_{\mathbf{gen}}(t) = a + bt^n \qquad (2)$$

in unit of $10^{14}/\mathrm{m}^2$, where n is ~ 0.43, according to the empirical defect generation theory, a and b are fitting parameters. The calculated net dislocation loop density, $\rho_{\mathrm{net}}(t) = \rho_{\mathrm{anni}} + \rho_{\mathrm{gen}}$, is shown by the solid (red) curve in Fig. 4b (Supplementary Figure 10). The simulation captures the essence of time-dependent evolution of mobile dislocation loop density, although the current model does not reflect details on the intermediate defect density states and does not consider the annihilation of mobile loops by other defect sinks. The details behind such cyclic variation of loop density require more comprehensive numerical modeling.

Supplementary References

1. Sadd, M. H., *Elasticity: theory, application, and numerics*. (Academic Press, 2014).
2. Silva, J. L. F. D. *et al.*, Convergedproperties of clean metal surfaces by all-electron first principles calculations. *Surf. Sci.* **600**, 703 (2006).
3. Wang, J. *et al.*, Detwinning mechanisms for growth twins in face-centered cubic metals. *Acta Mater.* **58**, 2262 (2010).
4. Sørensen, M. R. *et al.*, Diffusion mechanisms in Cu grain boundaries. *Phys. Rev. B* **62**, 3658 (2000).
5. Zhao, P. & Shimomura, Y., Molecular dynamics calculations of properties of the self-interstitials in copper and nickel. *Comput. Mater. Sci.* **14**, 84 (1999).
6. Zhu, Y. T. *et al.*, Dislocation–twin interactions in nanocrystalline fcc metals. *Acta Mater.* **59**, 812 (2011).
7. Li, N. *et al.*, Twinning dislocation multiplication at a coherent twin boundary. *Acta Mater.* **59**, 5989 (2011).
8. Yu, K. Y. *et al.*, In situ studies of irradiation-induced twin boundary migration in nanotwinned Ag. *Scripta Mater.* **69**, 385 (2013).
9. Zhu, Y. T. *et al.*, Deformation twinning in nanocrystalline materials. *Prog. Mater. Sci.* **57**, 1 (2012).
10. Wang, J. *et al.*, Dislocation structures of Σ3 {112} twin boundaries in face centered cubic metals. *Appl. Phys. Lett.* **95**, 021908 (2009).
